# Gene Transposition Causing Natural Variation for Growth in *Arabidopsis thaliana*

**DOI:** 10.1371/journal.pgen.1000945

**Published:** 2010-05-13

**Authors:** Daniela Vlad, Fabrice Rappaport, Matthieu Simon, Olivier Loudet

**Affiliations:** 1 Institut Jean-Pierre Bourgin, UMR1318 INRA-AgroParisTech, Versailles, France; 2 Institut de Biologie Physico-Chimique, UMR 7141 CNRS-UPMC, Paris, France; University of Georgia, United States of America

## Abstract

A major challenge in biology is to identify molecular polymorphisms responsible for variation in complex traits of evolutionary and agricultural interest. Using the advantages of *Arabidopsis thaliana* as a model species, we sought to identify new genes and genetic mechanisms underlying natural variation for shoot growth using quantitative genetic strategies. More quantitative trait loci (QTL) still need be resolved to draw a general picture as to how and where in the pathways adaptation is shaping natural variation and the type of molecular variation involved. Phenotypic variation for shoot growth in the Bur-0 × Col-0 recombinant inbred line set was decomposed into several QTLs. Nearly-isogenic lines generated from the residual heterozygosity segregating among lines revealed an even more complex picture, with major variation controlled by opposite linked loci and masked by the segregation bias due to the defective phenotype of SG3 (Shoot Growth-3), as well as epistasis with SG3i (SG3-interactor). Using principally a fine-mapping strategy, we have identified the underlying gene causing phenotypic variation at SG3: At4g30720 codes for a new chloroplast-located protein essential to ensure a correct electron flow through the photosynthetic chain and, hence, photosynthesis efficiency and normal growth. The SG3/SG3i interaction is the result of a structural polymorphism originating from the duplication of the gene followed by divergent paralogue's loss between parental accessions. Species-wide, our results illustrate the very dynamic rate of duplication/transposition, even over short periods of time, resulting in several divergent—but still functional—combinations of alleles fixed in different backgrounds. In predominantly selfing species like Arabidopsis, this variation remains hidden in wild populations but is potentially revealed when divergent individuals outcross. This work highlights the need for improved tools and algorithms to resolve structural variation polymorphisms using high-throughput sequencing, because it remains challenging to distinguish allelic from paralogous variation at this scale.

## Author Summary

Plant growth is a very complex character impacted by almost any aspect of plant biology and showing continuous variation among natural populations of a single species like *Arabidopsis thaliana*. Although difficult, it is important to reveal the precise genetic architecture of such a trait's variation to improve our understanding of the mechanisms and evolutionary significance of phenotypic variation. By using recombinant inbred lines derived from a cross between the reference strain ‘Col-0’ and the Irish strain ‘Bur-0’, we have localized several regions of the genome impacting plant growth. When attempting to confirm one of this region's effect, we revealed an even more complex genetic architecture where a first locus (which had remained undetected initially) has a major effect on growth only when a specific genotype was present at a second locus. We have shown here that the reason for this epistatic interaction between the two loci is that the functional allele for a gene important for photosynthesis efficiency and, consequently, growth, had been transposed from one locus to the other in Bur-0 compared to Col-0. This type of structural polymorphism seems to be frequent among strains and, although more difficult to detect, is likely to be of significant evolutionary importance.

## Introduction

Natural phenotypic variation observed among different genotypes (accessions, varieties, populations, etc) is partly explained by alterations of the genetic material. Identifying the molecular basis of phenotypic variation provides candidates to test how and where in the pathway adaptation is shaping natural variation [1]. Of particular interest is the type of sequence variation from which intra-specific diversity originates. In Arabidopsis for example, there is reason to suspect that along with single nucleotide polymorphisms and short indels, structural variants in the genome as well may be an important source of natural variation [2]–[5]. Structural submicroscopic variants (smaller than those recognized microscopically) are defined as genomic alterations (insertions, deletions, inversions, duplications and transpositions) involving segments of DNA ranging from the kb to the Mb scale [6]. They can occur in genomes after large segmental duplications and subsequent gene loss, or as the result of unequal or illegitimate recombination (tandem duplications/insertions, deletions/gene loss) [7], DNA segment inversions [8] or transposable elements activity (dispersed gene duplication) [9].

Little is known about the prevalence of this phenomenon in plants and its phenotypic consequences, but it was recently found to be widespread in yeast [10] and in humans [11] where large structural variants (>50 kb) are confirmed to affect ∼4,000 genomic loci among healthy individuals [12]. Two individuals are also estimated to differ at approximately 1,000 copy-number variations (CNVs) alone [13]. In humans, structural variation may have a more significant impact on phenotypic variation than SNPs and they were implicated in gene expression variation, female fertility, susceptibility to HIV infection, systemic autoimmunity, genomic disorders… [13], [14].

Structural variation may also contribute to postzygotic isolation through the production of genetically deficient hybrids [15], as recently demonstrated among Arabidopsis strains in a study describing how the transposition of the functional copy of an essential gene (balanced structural polymorphism) results in recessive embryonic lethality in an intraspecific cross [16]. Complex structural variation at the GS-Elong locus, including tandem *MAM*-gene duplication, gene loss, gene conversion and CNV was shown to cause natural variation for insect herbivore resistance [17]. Another example of natural variation for disease resistance in Arabidopsis was explained by the reciprocal loss of R-gene paralogues located in an ancient segmental duplication, which resulted in certain combinations lacking either functional copy [18].

Dispersed kind of structural variation will result in epistasis in intraspecific crosses (if the functional structural variation has phenotypic consequences) and could therefore be detected in mapping populations. Although experimental mapping populations of the type of recombinant inbred lines (RILs) allow detection of genetic interactions between loci, the number of RILs observed as well as possible segregation distortions caused by lethality or reduced fitness of particular genotypes may hamper the power to detect interacting QTL [19]–[21]. In this context, the residual heterozygosity existing in some RIL sets is a plus, since deleterious genetic combinations can be restored and studied from their ‘latent’ heterozygous states.

Currently, not much interest has been manifested for the detection and consequences of structural polymorphisms in plants, probably because it is even less convenient to detect and complement than ‘simple’ coding sequence changes in a gene, for example. Although the global impact of structural variation is unknown, it might have dramatic consequences on phenotypic diversity [22]. Unfortunately, array-based re-sequencing projects are limited to this respect as they can only easily detect deletions relative to the reference sequence [5]. For *A. thaliana*, the short sequence reads produced by deep-sequencing on Illumina proved to support, in addition to SNPs, the detection of short to medium-size indels, and the discovery of new sequences (absent from the reference genome) but not their sequence context [2]. Therefore, nothing is obvious about the frequency of structural variation and its association with phenotype as no ideal methodologies yet allow direct detection of structural variants in plants. Here we give an example of a functional structural polymorphism resulting from the divergent evolution of duplicate genes among *A. thaliana* accessions.

## Results/Discussion

We have used genome-wide molecular quantitative genetics to investigate natural genetic variation for shoot growth as a complex trait. Since the parental accessions were showing phenotypic differences with regard to shoot growth in our conditions, a subset of 164 Bur-0 × Col-0 Recombinant Inbred Lines optimized for QTL mapping [23] was grown and phenotyped *in vitro* in standard conditions in order to map loci affecting early stage shoot growth. Transgressive segregation of the shoot phenotypes observed among RILs (Figure S1A) indicates that the genetic potential for the study of shoot growth exists in this set. Indeed, four significant QTLs with LOD scores greater than 2.5 were mapped in this cross (Figure S1B).

### Confirming the chromosome 4 locus

**Figure 1:**
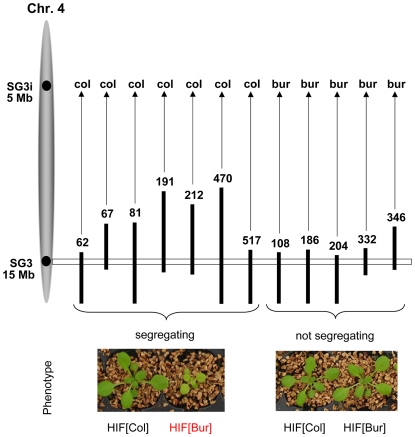
Confirmation of the phenotypic effect of SG3 and its interaction with SG3i. Black vertical bars represent heterozygous regions on chromosome 4 (represented in grey on the left) from different RILs used to generate HIFs and to check for the segregation of the defective growth/pale green phenotype. Photos of the plants were taken in the greenhouse three weeks after germination and show typical phenotypic segregation of individual HIFs. The phenotypic segregation of SG3 is conditioned by the presence of the Col allele at another locus: SG3i. The SG3i[Col]/SG3[Bur] combination causes a pale green pigmentation and defective growth.

In this work, we are now focusing on allelic variation in the genomic region underlying the QTL predicted between 14 and 15 Mb on chromosome 4. Confirmation of the phenotypic effect related to this locus was performed using specific NILs differing only for a small genomic region spanning a few cM around the QTL. NILs for this QTL were obtained by producing Heterogeneous Inbred Families (HIFs) which are easily generated taking advantage of the residual heterozygosity still segregating in F6 RILs [24], [25]. Initially, four candidate RILs (# 067, 081, 212 and 332), heterozygous only around the chromosome 4 locus, were used. In all HIFs except HIF332, the comparison of plants that were fixed for each parental allele (Col or Bur) at the QTL region revealed a highly significant difference in shoot growth (*P*<0.001). The HIFs[Bur] had marked phenotypic features (Figure 1) strongly reducing shoot size, as estimated either at a young stage *in vitro* (−25%) or at a later stage on soil (−70%), and chlorophyll content (−40% in greenhouse conditions). Then, bolting in plants carrying the Bur allele was delayed by approximately two weeks, plants were less robust with shorter inflorescence stems and yielded significantly fewer seeds than the HIFs[Col] plants, though flowers were normally developed and fully fertile. We named this QTL SG3 (Shoot Growth-3). Yet, the observed allelic effect was surprising: the Bur allele at the QTL was negatively affecting shoot size, while the QTL mapping had predicted that an opposite effect was segregating with this region (Figure S1). Moreover, HIF332 was not segregating for SG3 although its heterozygous region seemed to fully cover the QTL locus (and other positive HIFs' heterozygous regions), suggesting that the QTL is most probably involved in an epistatic interaction. The analysis of several (12) other independent RILs genotypically segregating at the locus revealed that SG3 was in complete interaction with a region at the top of chromosome 4, which we called SG3i (SG3-interactor; Figure 1). The phenotypic segregation of SG3 is conditioned by the presence of a Col allele at SG3i. Hence, with only two RILs (among our 164 subset) fixed for the combination of alleles giving rise to the defective growth phenotype, we could not have mapped this locus with the RILs and SG3 is a distinct locus than mapped initially in this region. Indeed, further analysis of HIFs genotypically segregating for the bottom of chromosome 4 region, but that do not segregate for SG3 (because harbouring a Bur allele at SG3i), showed that the locus mapped in the QTL analysis is real but distinct from SG3 and of much smaller–opposite–effect (HIF332; Figure S2). It is very likely that plants with the Col allele at SG3i and the Bur allele at SG3 (SG3i[Col]/SG3[Bur] combination) were unintentionally counter-selected during the single-seed descent process used for RIL fixation (despite the care to avoid any obvious bias) because of their retarded and small stature early after germination.

### Map-based cloning of SG3

**Figure 2:**
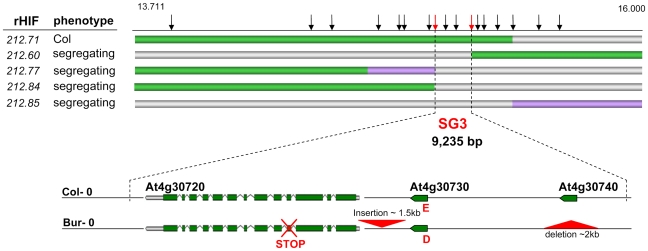
Fine-mapping of SG3. The genotype of the most informative recombinants (rHIFs) used to delineate the final 9 kb-candidate interval are represented with horizontal bars (green for Col allele, purple for Bur allele, grey for heterozygous). Vertical black arrows above represent frequently used markers for genotyping between 13.711 and 16.000 Mb on chromosome 4. Red arrows mark the most informative recombination breakpoints. Below, genes in the final candidate interval are illustrated for both parental accessions. Major polymorphisms are marked in red, including an E/D amino-acid change in At4g30730 and a premature STOP codon in At4g30720 in Bur-0.

We used HIF212 to further perform the fine-mapping of SG3 by recurrent (genotypic) selection and (phenotypic) analysis of recombinants within the target interval (rHIF, see Materials and Methods), as described earlier [26]. A first set of nine recombinants were identified in the initial heterozygous region of ∼5 Mb, when fixing HIF212. Each recombinant was tested for the segregation of the phenotype and the region of interest was fast reduced to ∼1 Mb. A following screen of 600 plants resulted in the isolation of 35 recombination events in the remaining interval. Recombinants with homozygous Bur-genotype at the QTL were easily phenotyped during the selection process (the Col allele is dominant at SG3) and seven other interesting recombinants were analysed by progeny-testing. Reliable phenotyping results allowed us to further narrow down the candidate interval to ∼100 kb. Finally, a last screen of 5,000 plants, descending from one positive rHIF, provided 34 additional recombinants in the 100 kb interval. The analysis of 12 interesting recombinants delineated the region of interest to a 9 kb-interval (Figure 2), precisely 9,325 bp between bordering polymorphisms internal to the recombination events. Based on the most informative recombinants (rHIF212.60 and rHIF212.77), an ‘advanced rHIF cross’ (arHIF; see Materials and Methods and [26]) was designed in order to obtain a line (arHIF212.97) that was segregating solely for the final candidate interval. This confirmed the presence of SG3 within the 9 kb-interval when comparing arHIF lines fixed for each allele (arHIF[Col] *vs* arHIF[Bur]). Three predicted genes (At4g30720, At4g30730 and At4g30740) are present in this interval.

The 9 kb-interval was sequenced in both parental accessions to identify putative functional polymorphisms. The presence of “heterozygous” (double) peaks in the sequence chromatograms when amplifying from Bur-0 DNA with primers within At4g30720, was taken as predictive of a duplication of (at least part of) the candidate region. SNP information was isolated from the “heterozygous” peaks, converted into CAPS markers and the duplication was mapped thanks to the original RIL set genetic map [23] between two markers at physical positions 5,629 and 6,923 kb, i.e. in the region of SG3i.

### Polymorphisms at SG3

Some major polymorphisms were identified in the SG3 candidate region when comparing Bur-0 to Col-0 (Figure 2). A ∼2 kb region including At4g30740 is absent from the Bur-0 accession. Conversely, a large (1,130 bp) insertion is present in Bur-0, 180 bp upstream of At4g30720 (it includes LTR and ADN/MuDR transposon traces and a putative target site duplication TGATG/TGATG). Also, a 1 bp-deletion in exon four of At4g30720 results in a frame shift, predicting a premature stop codon which terminates the ORF after 5 amino acids. Finally, one non-synonymous SNP is present in the coding sequence of At4g30730, changing glutamic acid (E)#66 into aspartic acid (D).

### Identification of the causal gene

The predicted At4g30720 is encoding a 707aa-putative oxidoreductase/electron carrier detected in the chloroplast stroma [27]. It has a predicted FAD-dependent oxidoreductase domain (IPR006076), adrenodoxin reductase domain (IPR000759) and N-terminal domain (www.arabidopsis.org; www.ncbi.nlm.nih.gov). Both At4g30730 and At4g30740 putatively encode very small proteins of unknown functions, if not pseudogenes (both are partially matching neighbouring genes, respectively At4g30750 and At4g30710, and they are not supported by ESTs and expression data).

**Figure 3:**
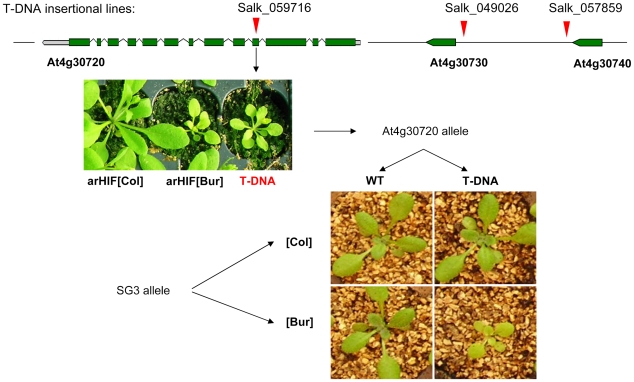
Quantitative complementation of an At4g30720 T–DNA allele. T-DNA insertion points are marked by red arrows. SALK_049026 hits the promoter of At4g30730 and SALK_057859 hits the 3′UTR of At4g30740 and have no phenotypic consequences in the Col-0 background. The phenotype of the SALK-059716 T-DNA homozygous mutant (an insertion in the fourth exon of At4g30720) is shown below and strikingly resembles the arHIF[Bur] phenotype. The complementation test combined the QTL alleles ([Col] or [Bur]) with either a mutant (T-DNA) or a wild type (WT, i.e. Col) allele at the candidate gene. Typical phenotypes resulting from the 4 combinations are shown and revealed that the SG3[Bur]-allele fails to complement the mutant phenotype.

T-DNA insertions in At4g30730 (SALK_049026-promoter) and At4g30740 (SALK_057859-3′UTR) had wild-type phenotypes for shoot growth. Instead, a homozygous mutant line (SALK_059716) with a T-DNA insertion in the fourth exon of At4g30720 in a Col-0 background, phenotypically resembled plants with SG3i[Col]/SG3[Bur] allelic combination (Figure 3). In addition, RT-PCR assays using primers annealing to the exon sequences flanking the T-DNA insertion site failed to amplify any full-length transcript, indicating that this mutant is very likely a knock-out.

Quantitative complementation tests showed that the Col allele at SG3 was able to rescue the mutant allele phenotype, while, in an identical F1 genetic background, the Bur allele failed to complement the T-DNA allele (Figure 3). This indicates that the phenotype observed in arHIF[Bur] is probably due to a defect in At4g30720 and that (in the absence of any observed expression-level differences) the 1 bp-deletion found in At4g30720[Bur] is likely to be the causal polymorphism.

### Characterizing SG3i

A Bur-0 BAC genomic library was used to sequence the duplicated copy of At4g30720 at SG3i. Although a non-synonymous SNP is causing an amino-acid change compared to At4g30720[Col], converting alanine #43 into threonine, it seems that this copy must be encoding a functional protein and explains why the Bur-0 accession itself does not have a small, pale green phenotype. Our sequencing results on the BAC identify a 10 kb-region (including At4g30720' paralogue) at SG3i clearly corresponding to the SG3 region surrounding At4g30720. Efforts have been made but we did not manage to obtain any good sequence (corresponding to any known stretch of DNA that would correspond to our reference sequence, Col-0) when sequencing from the 10 kb duplicated region toward the outside. Direct sequencing of the BAC was then impossible due to double priming which suggests the presence of repetitive DNA around the duplicated region. In addition, attempts to amplify specific loci (especially genes neighbouring the 10 kb duplicated region from SG3, like At4g30710, At4g30750, …) from the SG3i BAC gave no results, so we consider likely that the paralogues arose from a small-scale dispersed duplication event.

### Genetic mechanism

**Figure 4:**
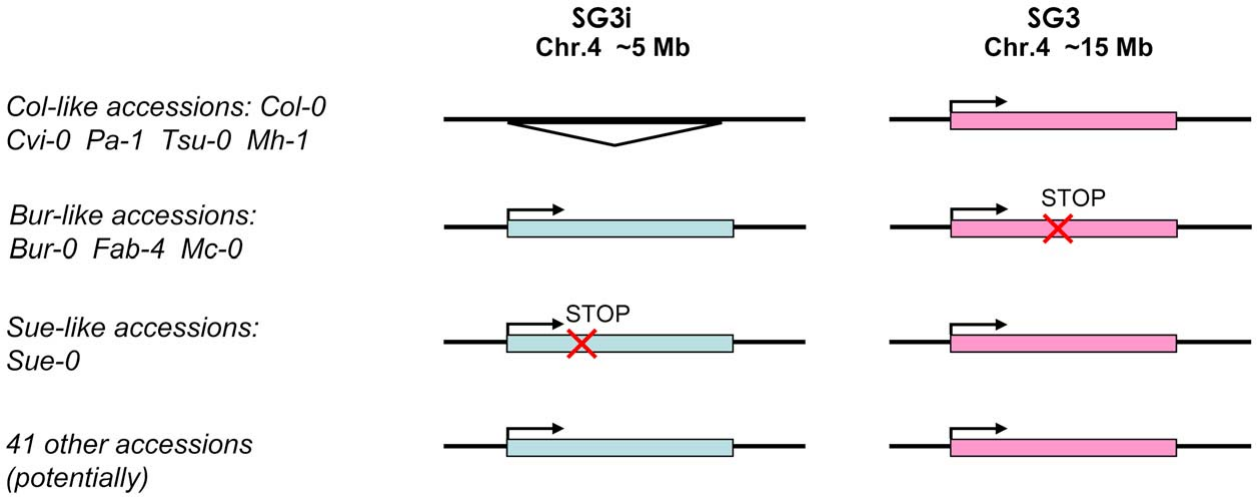
Species-wide patterns of diversity for the SG3/SG3i duplication of At4g30720. Accessions are grouped according to the divergence of the duplicate gene pair schematized on the right. A triangle indicates the complete absence of the paralogue. The ‘stop’ mark indicates the presence of a premature stop codon. The majority of accessions fall in the last group where no major functional polymorphism has been detected for both copies.

Overall, our results support the following: At4g30720 is responsible for the observed phenotype; two copies are present in the Bur-0 genome, one of which is not functional (at SG3); only one (functional) copy is present in the Col-0 genome (at SG3). Thus, plants that are homozygous for the Col allele at the SG3i locus and at the same time homozygous for the Bur allele at SG3, have no functional copy of At4g30720, resulting in the defective growth phenotype (Figure 4). This theory is supported by the mutant line SALK_059716 which represents an equivalent combination of alleles and by the observation of the segregation of the phenotype in other independent crosses (see below). We suggest that the transposition of the functional copy of the gene in Bur-0 (compared to Col-0) is the result of divergent evolution of the paralogues after an ancestral (dispersed) single gene duplication. This type of structural variation could have been previously underestimated as it has no phenotypic impact on the parental accessions themselves but seems to evolve quickly and could therefore represent a major source of intraspecific diversity and constraints [16].

### Species-wide diversity

We sequenced ∼3,400 bp of At4g30720 from 52 different accessions (essentially our core-collection of 48 [28]; Table S1) to investigate the species-wide patterns of diversity for this duplication event. The SG3i amplicons were distinguished from SG3 by a specific 7 bp-indel located 125 bp upstream of the start codon. The vast majority of accessions analysed bear two copies, while only 5 have a single copy, like Col-0. Most of the two copies-accessions seem to have both copies functional (no obvious functional polymorphisms in the coding sequence). Bur-0, Mc-0 and Fab-4 share the same polymorphism resulting in a premature stop codon in At4g30720, while Sue-0 presents a distinct 1 bp-indel causing a premature stop codon in the copy present at SG3i (Figure 4). RT-PCR analyses were performed for a subset of 26 accessions. In all two copies-accessions tested, transcripts were detected from both paralogues, with no exceptions.

In addition, crosses between Bur-like and Col-like accessions were designed in order to independently validate the candidate genetic mechanism and the functional group in which accessions belong (Figure 4). The F2 progeny of a cross between the single copy-accession Cvi-0 and Bur-0 is clearly segregating for the small, pale green phenotype in accordance with segregation of alleles at SG3/SG3i loci, indicating that Cvi-0 has a functional copy of At4g30720 at SG3 despite bearing a haplotype much closer to Bur-0 than to Col-0 at this locus (see below). Also, the F2 progeny of reciprocal crosses between Col-0 and either Mc-0 or Fab-4 (both Bur-like accessions) confirmed that a similar allelic combination is present in these Bur-like accessions. As expected, segregation of the phenotype was also observed in the reciprocal crosses between Sue-0 and Bur-0, in agreement with our sequencing results. In each case the causality link between SG3/SG3i allelic combination and the phenotype was confirmed by genotyping at these two loci. As negative controls, no phenotype was observed in the Sue-0 × Col-0, Cvi-0 × Col-0 reciprocal crosses' progeny, as well as in crosses between Bur-0 and other Bur-like accessions. The clear link observed between the segregation of diverse functional alleles at SG3/SG3i and the phenotype in distinct unrelated genetic backgrounds is another strong confirmation of the identity of the QTL.

**Figure 5:**
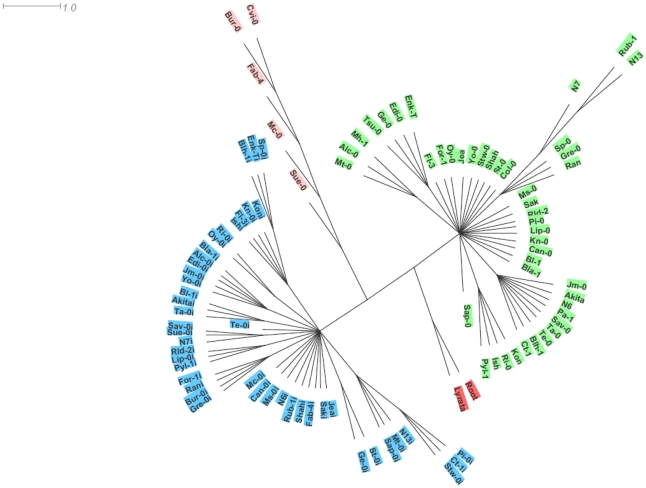
Phylogenetic analysis of the paralogues. A rooted haplotype network is shown, generated from the genomic locus sequences and using *A. lyrata* as outgroup. To distinguish SG3i copies, the names of accessions are followed by an ‘I’ for this paralogue. SG3i copies all cluster together and form a first haplotype group (highlighted in blue). A second cluster (highlighted in green) groups most of the SG3 copies, with the exception of Bur-0, Fab-4, Mc-0, Sue-0 and Cvi-0 which branch together as a third cluster (highlighted in pink).

Phylogenetic analysis, using *A. lyrata* as outgroup, essentially clustered the two paralogues in distinct haplotype groups. The SG3 cluster, contains most of the SG3 copies except for those from Bur-0, Fab-4, Mc-0, Sue-0 and Cvi-0 which branch together as a divergent group with a distinct haplotype (Figure 5). Overall, each of the three clusters have distinctive polymorphisms and we see no obvious genealogy between the two main clusters and either the minor cluster or *A. lyrata* (Table S2). It seems that the minor cluster evolved, in some way, away from the rest of the SG3 copies. Ectopic recombination and gene conversion (the extents of which remain to be shown in plants) could also participate in generating new haplotypes during duplicated gene evolution [29]. Very likely, the At4g30720 alleles found in the minor cluster derive from a common ancestor. It is nevertheless surprising to see the very divergent path followed by the five accessions bearing this haplotype, with at least one belonging to each of the first three functional groups depicted on Figure 4. The Bur-0, Fab-4 and Mc-0 SG3 copies lost their function (premature stop codon) probably very recently; while the Cvi-0 and Sue-0 copies maintained undifferentiated functions (as confirmed in the crosses) and are found associated with, respectively, a deleted and a non-functional paralogue at SG3i. Hybridization between accessions bearing different alleles could explain the diversity of allelic combinations detected at these unlinked loci.

As found previously [16], we saw no clear correlation between our haplotype network and the population structure described on a very similar accession sample [30].

### Molecular population genetics

#### A recent duplication

In a similar fashion as described by Moore and Purugganan [31], i.e. based on Ks–the number of silent substitutions per site between duplicate gene pairs–and a molecular clock calibrated with an *A. thaliana*/*A. lyrata* divergence date 5.2 million years ago (mya) [32], we estimate the date of duplication of our paralogues to be about 0.25+/−0.035 mya. We conclude that the two copies of At4g30720 at SG3 and SG3i arose from a duplication event that occurred late after the divergence of *A. thaliana* from its close relative *A. lyrata* and before the wide recent colonization event/population expansion [33].

It was not possible to clearly infer the progenitor locus from the haplotype network generated from At4g30720 and its paralogue's sequences rooted with *A. lyrata* (Table S2). However, conserved synteny between the region of SG3 in *A. thaliana* and the region of the unique copy of this gene in *A. lyrata* suggests that SG3 is the progenitor locus. As described above, it seems that this duplicate gene pair would then be the result of a small-scale duplication of ∼10 kb.

#### Tests of selection

According to our estimates, total sites nucleotide diversity, θπ, varied between 0.0009 and 0.0019 for the paralogues at SG3 and SG3i respectively, consistently lower than the nucleotide diversity observed for the average Arabidopsis nuclear gene (0.0071; [34], [35]). Nevertheless, the copies at SG3i are overall more polymorphic than the ones at SG3, indicating that they may have evolved more rapidly (Table S3). To test if the reduced level of nucleotide polymorphism observed in both copies is associated with an hypothetical directional selection event, we performed a multilocus HKA test [36]: we compared sequence polymorphism and divergence at SG3 to four reference loci previously described as neutral (*COI1*, *EDR1*, *NDR1* and *TGA2*; [37]). The results showed no significant difference (*Chi2* = 4.715; *P* = 0.3178), indicating that our genes have levels of variation that do not differ from those of neutral reference loci. For SG3, both Tajima's D (D = −2.0506) and Fu and Li statistics D* (D* = −3.1489) indicate an excess of rare polymorphisms (singletons), significantly deviating from expectations based on the neutral-equilibrium model of molecular evolution [38], [39]. But this excess of singletons, also described at the genome scale [35], could simply reflect the inbreeding nature of the plant or a rapid post-Pleistocene expansion [40]–[42].

### Toward the role of At4g30720

**Figure 6:**
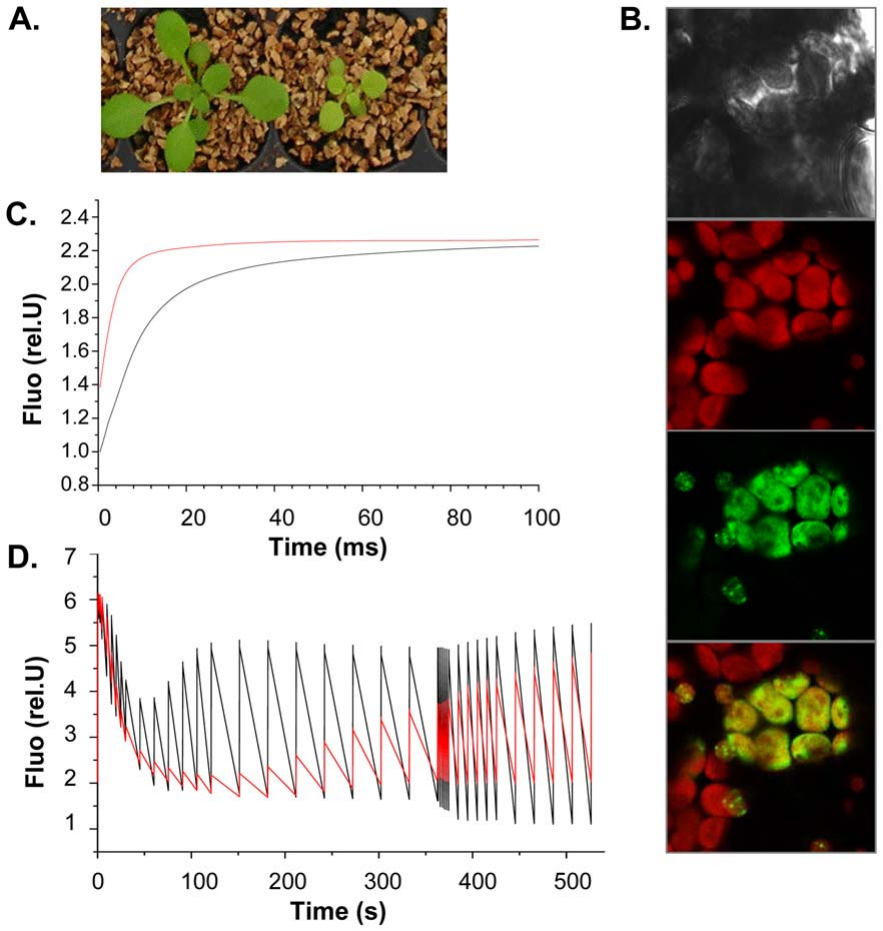
SG3 affects the photosynthetic electron transfer rate with consequences on shoot growth. (A) Severe consequences of SG3 on pigmentation and growth observed three weeks after sowing in arHIF[Bur] (bottom) compared to arHIF[Col] (top). (B) Transient expression in Arabidopsis cotyledons of the At4g30720-GFP fused target peptide showing the localization of the protein into the chloroplast, as shown by colocalization with the chloroplast autofluorescence in red. (C) Light-induced fluorescence changes in the presence of DCMU (inhibitor of PSII) suggesting that the overall PSII + PSI content is likely to be decreased in arHIF[Bur] (red) compared to arHIF[Col] (black). (D) Differences in fluorescence observed between arHIF[Col] and arHIF[Bur] under continuous illumination. A strongly quenched F_max_' and its delayed recovery suggest a severely delayed activation of the Benson-Calvin cycle and a decrease in the electron flow through the photosynthetic chain in arHIF[Bur].

Phenotypically, at the vegetative stage, SG3[Bur] plants are always pale green, with fewer leaves and rosettes about 70% smaller than the SG3[Col] plants (Figure 6A). SG3[Bur] plants contain less chlorophyll (a and b) and the chlorophyll a/chlorophyll b ratio is significantly modified (Table S4).

Transient expression assays of the GFP-fused (N-terminal) target peptide in Arabidopsis cotyledons confirmed the exclusive localization of the protein into the chloroplast (Figure 6B).

Chlorophyll fluorescence studies were undertaken to find evidence of the physiological role of At4g30720. Light-induced absorption changes in the absence/presence of DCMU and hydroxylamine (PSII inhibitors) at 520 nm measured 100 µs after a single turnover flash was employed to quantify the PSII/PSI stoichiometry in arHIFs. The PSII/PSI ratio seems not to be altered in the arHIF[Bur] compared to the arHIF[Col]. However, the arHIF[Bur] showed a lower F_max_/F_0_ ratio suggesting a higher F_0_ and, hence, a significant amount of PSII antenna not being connected to the PSII photochemical trap (Figure 6C). Together with the unaltered PSII/PSI stoichiometry, these data suggest that the overall PSII + PSI content is likely to be lower in arHIF[Bur].

In line with this hypothesis, the fluorescence changes observed during an illumination of a few minutes show that the line with a Bur allele at SG3 is marked by strongly quenched F_max_' and that the recovery of this quenching is severely delayed (Figure 6D). This may reflect the delayed activation of the Benson-Calvin cycle which would stem from the decrease in the electron flow through the photosynthetic chain.

We tend to think that it is not a specific effect on one of the photosynthetic complexes because the PSII/PSI stoichiometry is not affected. Rather, by one way or another, the main consequence on the photosynthetic chain is a decrease in the number of complexes involved in electron transfer, resulting in an overall impaired CO_2_ assimilation. The resulting defect in carbon metabolism could justify the delay in growth observed in the arHIF[Bur] and T-DNA mutant.

### General conclusion

Our work highlights the very dynamic rate of evolution of duplicate genes in Arabidopsis where multiple divergent–but still functional–combinations of alleles can be fixed in different backgrounds over a limited period of time. Even genes essential for fundamental processes, like photosynthesis here, can be affected. In the present work, just as in previously described examples from Arabidopsis [16], [18], the structural variation has its origin in a duplication event followed by paralogue's extinction, a loss that can occur early in the process of duplicate gene evolution [31]. Due to the numerous large-scale segmental duplications and dispersed small-scale gene duplications, we can expect a high prevalence of this phenomenon in plants, potentially significantly impacting and constraining phenotypic variation generated at the intraspecific level.

The real extent of structural variation remains to be evaluated and sequencing projects at the species level are willing to consider this [22]. However, to enable a more comprehensive detection of structural variation contributing to intraspecific genetic diversity, significantly longer reads and paired-end sequencing–at the least–are necessary [11], [43] as well as new algorithms to analyse this data [44]–[46]. This should reveal at least CNV polymorphisms, but an extreme case of structural variation is balanced gene transposition which remains challenging to solve because it is then difficult to distinguish allelic from paralogous variation [10]. In Arabidopsis species-wide sequencing studies, one should expect to commonly face new DNA sequences, for which we have no reference and/or no idea of the insertion context, as it is clear that most Arabidopsis accessions have genome sizes 5 to 10% larger than the reference Col-0 genome [3]. A true *de novo* assembly of high-quality complete Arabidopsis genomes should elucidate all types of polymorphisms that can dictate natural variation.

## Materials and Methods

### Plant material

A subset of 164 Bur-0 × Col-0 RILs (http://dbsgap.versailles.inra.fr/vnat/; [23]) optimized for QTL mapping was grown *in vitro* on standard media and phenotyped to map QTLs affecting early-stage shoot growth (see below). RILs 067, 081, 212, 332 that still segregated only for a limited region around 15 Mb on chromosome 4 were used to generate HIFs [24], which enabled the comparison of lines containing either of the parental alleles at the locus of interest in an otherwise identical background. The progeny of RIL212 was genotypically screened to find recombinants used in the fine-mapping process (see below). All (22) lines in the complete RIL population that were still heterozygous around 15 Mb were analysed by progeny-testing to identify and roughly map an interactor controlling SG3 phenotypic segregation. T-DNA insertion lines at At4g30720 (SALK_059716), At4g30730 (SALK_049026) and At4g30740 (SALK_057859) were ordered from NASC and grown under greenhouse conditions. The 52 accessions used to explore species-wide diversity at SG3/SG3i are listed and described in Table S1. They represent most of the core-collection of 48 accessions from the Versailles Center for Biological Resources ([28]; http://dbsgap.versailles.inra.fr/vnat/), plus a few more references and specifically selected accessions.

### Shoot measurements

Seeds were surface-sterilized by soaking 10 minutes in 70% EtOH, 0.1% TritonX-100, followed by one wash with 95% EtOH for another 10 minutes. Under sterile conditions, the seeds were suspended in a 0.1% agar solution and stratified in the dark at 4°C for 4 days. Then, seeds were sown on square Petri dishes (120 mm) containing classical Arabidopsis media [47], with 9 RILs per plate and 9 seeds representing each RIL. Plants were grown for 11 days in a culture room (21°C, 16 hours light/8 hours dark cycle) where plates were rotated daily. At 10 DAG (days after germination) plantlets were carefully flattened onto the surface of the media and scanned on a flatbed scanner. Projected shoot area was measured with Optimas 6.5 as an estimate of shoot growth.

### QTL mapping

To find genetic loci that affect trait variation, average shoot areas were used as quantitative values to carry out a simple interval mapping using the WebQTL tool (www.genenetwork.org). A 1000 permutations-test was used to estimate a significance threshold at 2.5 LOD. Scanning of the genome with 2 loci mapped simultaneously including their interaction (pair-scan) was performed to search for complex epistasis between pairs of loci that could explain the trait variation.

### Fine-mapping

Phenotyping for the confirmation of the QTL segregation during the fine-mapping process was performed as described above, except that seeds were not surface-sterilized and plants were grown in the greenhouse on soil. For each RIL, 48 plants were grown and genotyped to isolate individuals fixed for the parental alleles in the interval (HIF) as well as possible recombinants within the heterozygous region (rHIF). For the chosen HIF (HIF212), more recombinants were searched in two successive screens of respectively 600 and 5,000 plants. Genotyping during screens involved microsatellite or indel markers to identify recombination events within the candidate region. Once recombinants had been identified, CAPS markers and direct sequencing were used to refine and localize recombination breakpoints to smaller intervals when needed. Interesting (informative) rHIFs were then tested for the segregation of the defective growth/pale green phenotype by progeny-testing.

Advanced rHIF line 212.97 that segregate solely for the candidate region (and, hence, for the phenotype) was obtained from crosses between two different rHIFs lines with adequate genotypes (rHIFs recombined immediately to the north or immediately to the south of the SG3 final interval and with adequate genotype elsewhere), following a strategy initiated earlier [21] and as described by Loudet *et al.* [26].

### Allelic complementation

F_1_ plants used for the quantitative complementation assay were generated by reciprocally crossing heterozygous arHIF212.97 to the heterozygous T-DNA insertion line, SALK_059716. F_1_ plants were phenotyped and genotyped to detect which allelic combination at SG3 was restoring the wild-type phenotype or not.

### Sequencing At4g30720 paralogues

Bur-0 SG3 and SG3i copies were sequenced using a BAC library (Amplicon Express). Corresponding BACs were selected by PCR using primers for IND414975 amplifying a fragment of ∼1,300 bp at SG3 and respectively ∼100 bp at SG3i. BAC DNA was then extracted with the NucleoBond BAC 100 kit (Macherey-Nagel) and sent for sequencing.

For the sequencing of the 52 accessions, a specific primer (INDEL'75R) based on an indel polymorphism was used to specifically amplify a 4.5 kb-fragment covering the SG3i copy of the gene. The obtained PCR product was used as template to further PCR amplify and sequence five overlapping fragments. We inferred the SG3 sequence from an unspecific sequencing reaction by manually subtracting the SG3i polymorphisms (according to the specific SG3i sequence above), assuming none of our accessions had residual heterozygosity at these loci (which is extremely likely after the numerous SSD cycles they have been through). Primers used for sequencing are listed in Table S5. *A. lyrata* sequence was obtained from the Joint Genome Institute sequencing project data (http://genomeportal.jgi-psf.org/Araly1/Araly1.home.html).

### At4g30720 expression studies

At4g30720 expression in the transgenic plants was analyzed by RT-PCR. RNA was extracted with the RNeasy Plant Mini Kit (Qiagen) and the reverse transcription performed with the RevertAid First Strand cDNA Synthesis Kit (Fermentas) to yield single-strand cDNA. Transcription was tested with the following primers, forward primer 5′-CGTTTTCAACACCGCTAGAAC-3′ and reverse primer 5′- TGGTTTGTTTGCTGCTCTTG-3′, flanking the T-DNA insertion site. The adenine phosphoribosyl-transferase gene was used as control in the RT-PCR reaction.

Accessions were grown in vitro for 2 weeks then shoots were frozen and ground in liquid nitrogen. RNA was extracted and reverse-transcribed as above. PCR was carried out with the following primers, forward primer 5′- TGGTTTGTTTGCTGCTCTTG -3′ and reverse primer 5′- AACAACACGAGAATCCTCTACCA -3′, complementary to both SG3 and SG3i copies in all accessions. Amplicons generated (410 bp) were digested with BclI (Fermentas). Due to a SNP in the sequence shared by all SG3i copies among accessions tested, SG3i amplicons are digested while SG3 amplicons remain undigested, allowing to distinguish each paralogue's expression.

### Molecular population genetics data analysis

All accessions' sequences were aligned using CodonCode Aligner v3.01 and manually checked for all polymorphisms. The haplotype network was generated using Splitstree4 V4.10 on all sequenced accessions [48]. Population genetics analyses were generated on a subset of 41 accessions with both SG3 and SG3i functional copies, and *A. lyrata* ortholog was used as the outgroup in the analyses. Estimates of nucleotide diversity θπ, nucleotide divergence (Ks), Tajima's D and Fu et Li D* were performed using DnaSP5 [49]. The multilocus HKA test was performed on silent sites using a program kindly provided by J. Hey (Rutgers University, Piscataway, NJ).

### Chlorophyll fluorescence

The fluorescence changes were measured as described previously [50]. The fluorescence changes were induced by a continuous green light (100 µE.m^2^.s^−1^) allowing the measurement of the fluorescence yield of the dark-adapted sample (F_0_) and the fluorescence yield reached under quasi steady-state conditions (F_stat_). The maximum fluorescence yield was measured 50 µs after applying an intense (5,000 µE.m^2^.s^−1^) light-pulse of 200 ms duration.

## Supporting Information

Figure S1 (A) Frequency distribution of shoot growth phenotypes. Distribution of the shoot growth phenotypes for a set of 164 Recombinant Inbred Lines (RILs) derived from the Bur-0 × Col-0 cross, grown in standard in vitro conditions. The y-axis represents the number of lines for each phenotype class (x-axis, in pixel per plant). The parental phenotypes are indicated in red. (B) Interval mapping results for shoot growth in the Bur-0 × Col-0 RIL population. The horizontal axes represent the Arabidopsis genome, each separate section corresponding to a chromosome as indicated above graphs. The thick blue curve represents the statistical significance of the QTL (LOD Score; scale on the left). Pink and grey horizontal lines respectively show the significant and suggestive thresholds for QTL detection. Thinner red/green curve represents the estimated allelic effect of the hypothetical QTL at each location, when respectively Col- or Bur-alleles increase the trait value (scale on the right, in trait units).
(0.07 MB PDF)

Figure S2 Masked QTL effect in HIF332. Mean rosette area (in pixel per plant) of individuals from HIF332 fixed for the Col (HIF332-Col) or Bur (HIF332-Bur) allele at the QTL region. Different letters on bars indicate significantly different means (*P*<0.01). HIF332 is not segregating for SG3.
(0.01 MB PDF)

Table S1 Accessions (n = 52) used to explore the species-wide diversity at At4g30720 and paralogue.
(0.03 MB XLS)

Table S2 Haplotype groups at At4g30720 (SG3) and paralogous locus (SG3i).
(0.09 MB XLS)

Table S3 Polymorphism analysis at the SG3 and SG3i loci.
(0.02 MB XLS)

Table S4 Chlorophyll contents and ratio, from different SG3 genotypes (means and standard error of the means).
(0.02 MB XLS)

Table S5 Primers used for sequencing At4g30720 in the 52 accessions.
(0.02 MB XLS)
