# Supplementary figures and images for: Gene Transposition Causing Natural Variation for Growth in Arabidopsis thaliana
Source: PLoS Genet. 2010 May 13;6(5):e1000945. doi: 10.1371/journal.pgen.1000945 (PMC2869320; doi:10.1371/journal.pgen.1000945)

Figure S1

A

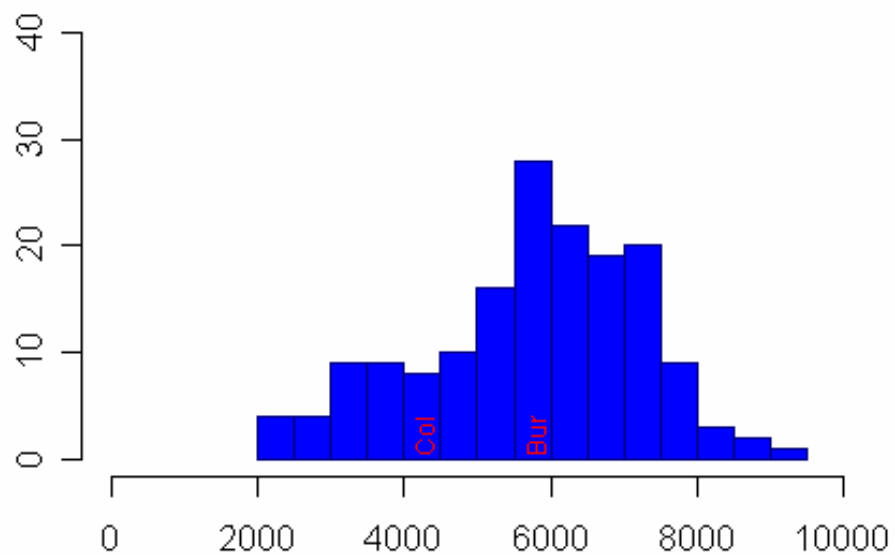

B

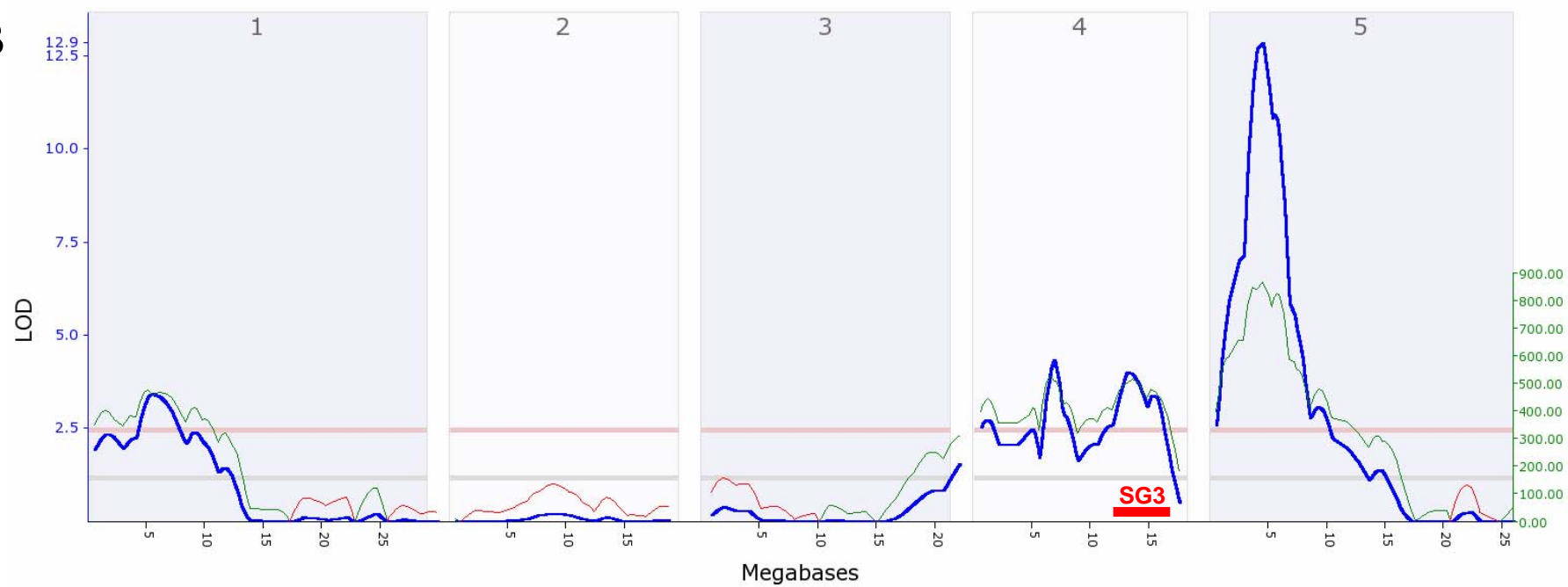

Supplement: Figure S1 — (A) Frequency distribution of shoot growth phenotypes. Distribution of the shoot growth phenotypes for a set of 164 Recombinant Inbred Lines (RILs) derived from the Bur-0 × Col-0 cross, grown in standard in vitro conditions. The y-axis represents the number of lines for each phenotype class (x-axis, in pixel per plant). The parental phenotypes are indicated in red. (B) Interval mapping results for shoot growth in the Bur-0 × Col-0 RIL population. The horizontal axes represent the Arabidopsis genome, each separate section corresponding to a chromosome as indicated above graphs. The thick blue curve represents the statistical significance of the QTL (LOD Score; scale on the left). Pink and grey horizontal lines respectively show the significant and suggestive thresholds for QTL detection. Thinner red/green curve represents the estimated allelic effect of the hypothetical QTL at each location, when respectively Col- or Bur-alleles increase the trait value (scale on the right, in trait units). (0.07 MB PDF) [file pgen.1000945.s001.pdf]

# Figure S2

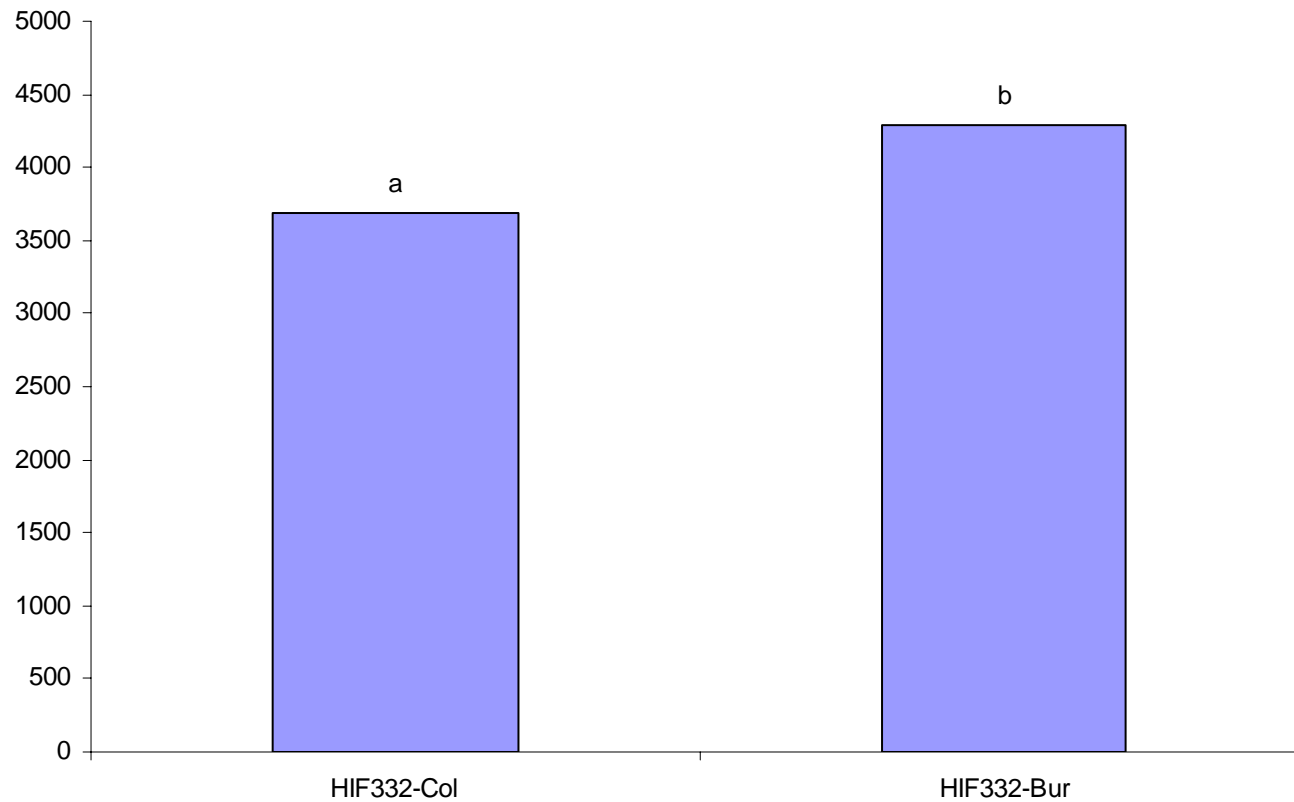

Supplement: Figure S2 — Masked QTL effect in HIF332. Mean rosette area (in pixel per plant) of individuals from HIF332 fixed for the Col (HIF332-Col) or Bur (HIF332-Bur) allele at the QTL region. Different letters on bars indicate significantly different means (P<0.01). HIF332 is not segregating for SG3. (0.01 MB PDF) [file pgen.1000945.s002.pdf]
